# Clinical Predictors of Progressive Hemorrhagic Injury in Children with Mild Traumatic Brain Injury

**DOI:** 10.3389/fneur.2017.00560

**Published:** 2017-11-13

**Authors:** Guangfu Di, Hua Liu, Xiaochun Jiang, Yi Dai, Sansong Chen, Zhichun Wang, Hongyi Liu

**Affiliations:** ^1^Department of Neurosurgery, Yijishan Hospital, Wannan Medical College, Wuhu, China; ^2^Department of Neurosurgery, The First People’s Hospital of Kunshan, Jiangsu University, Suzhou, China; ^3^Department of Neurosurgery, The Affiliated Brain Hospital, Nanjing Medical University, Nanjing, China

**Keywords:** mild traumatic brain injury, children, progressive hemorrhagic injury, computed tomography, clinical predictors

## Abstract

**Objective:**

Traumatic brain injury (TBI) occurs commonly in children. Repeat computed tomography (CT) follow up of TBI patients is often scheduled to identify progressive hemorrhagic injury (PHI). However, the utility of repeated CT scans, especially in children with mild TBI [Glasgow Coma Scale (GCS) scores of 13–15], has been debated. The purposes of the present study were to identify clinical predictors of PHI in children with mild TBI and to clarify relevant clinical factors *via* radiological examination.

**Methods:**

From 2014 to 2016, we retrospectively enrolled children <15 years of age with mild TBI. We recorded age, sex, GCS scores on admission, causes of head injury, timing of initial CT, any loss of consciousness, vomiting and seizure data, and type of TBI. Based on repeat CT findings, patients were dichotomized into either a PHI group or a non-PHI group. Also, clinical data were comparatively reviewed. Multivariate logistic regression analysis was used to identify clinical predictors of PHI.

**Results:**

Of the 175 enrolled children, 15 (8.6%) experienced PHI. Univariate analysis revealed that GCS score on admission, cause of head injury, vomiting, seizure, and TBI type were associated with PHI. Multivariate logistic regression analysis showed that a GCS score of 13 and epidural hemorrhage (EDH) were independently associated with PHI (hazard ratio = 0.131, *P* = 0.018; hazard ratio = 6.612, *P* = 0.027, respectively).

**Conclusion:**

A GCS score of 13 and EDH were associated with PHI. These factors should be considered when deciding whether to repeat CT on children with mild TBI.

## Introduction

Traumatic brain injury (TBI) is common in children and is an important cause of disability and morbidity (1). The incidence of TBI in children is estimated to be 250 per 100,000 per year. TBI accounts for >7,000 deaths and 600,000 emergency department visits annually among children in the United States (2). The vast majority of TBIs in children are mild, requiring no specific therapy, and associated with no sequelae (1). However, a small proportion of patients presenting with mild TBI develop progressive hemorrhagic injury (PHI). Therefore, it is important to recognize and intervene early in PHI to improve prognosis. Computed tomography (CT) scanning for initial evaluation of TBI is well-established. Repeat CT is also frequently performed. However, neither the indications for, nor the timing of, repeat CT scans in children are well-established. Most centers routinely schedule CT for patients with moderate or severe TBI, but the utility of such an approach remains debatable in patients with mild TBI, especially children. Studies advocating routine repeat CT suggest early medical intervention *via* osmotic therapy or surgical intervention featuring placement of an intracranial pressure monitor or craniotomy before neurological deterioration is evident (3–5). However, several recent studies have questioned this practice, rather advocating the use of repeat CT only for non-examinable patients and those exhibiting no improvement on neurological examination (6, 7). Children are more vulnerable to the carcinogenic effects of radiation than are adults because of their higher cell replication rates and their longer expected lifespans. It is well-known that the younger the age at the time of radiation exposure, the higher the risk of cancer induction (8, 9). Minimizing the number of CT scans is of great importance in children, because of the increased risk of lethal malignancies induced by exposure to ionizing radiation (10, 11). No present guideline addresses the utility of repeat CT in terms of the follow up of mild TBI in children. The purposes of this study were to assess the predictive role of clinical factors on admission in children with mild TBI using sequential radiological examination.

## Materials and Methods

This retrospective study was approved by the Institutional Ethics Board of the Yijishan Hospital of Wannan Medical College. All medical records of children <15 years of age who had been hospitalized for mild TBI between January 1, 2014 and December 30, 2016 were reviewed. Data collected included age, sex, Glasgow Coma Scale (GCS) score on admission, causes of head injury, timing of initial CT, any loss of consciousness (LOC), vomiting and seizure status, and type of TBI. Inclusion criteria were as follows: (1) GCS score on admission ≥13; (2) initial head CT within 24 h after trauma; and (3) at least one repeat CT scan within 72 h of admission. Exclusion criteria were as follows: (1) any known coagulation disorder; (2) severe thoracic or abdominal injury; (3) diagnosis of a skull-base fracture based on clinical symptoms; and (4) initial cranial CT scan revealing the need for emergency surgery. PHI was diagnosed if the repeat CT scan showed worsening of the condition because of development of a new lesion or an increase ≥25% of the original lesional volume as shown on the first post-injury CT scan (4). We divided all patients into a PHI group and a non-PHI group.

All data were analyzed using SPSS (SPSS, Inc., Chicago, IL, USA) software. The χ^2^ test was employed to compare differences in categorical outcome variables between the two groups. We used the independent-samples *t*-test to compare differences between continuous parametric variables and the Mann–Whitney *U* test to compare differences between non-parametric continuous variables. Continuous parameters are shown as mean ± SD. After identifying risk factors for PHI *via* single-factor analysis, we further performed logistic regression analysis to determine predictors of PHI. Such predictors were deemed significant if the probabilities that they were insignificant were less than 0.05.

## Results

From January 1, 2014 to December 30, 2016, 175 children <15 years of age who presented to Yijishan Hospital of Wannan Medical College with mild TBI met the inclusion criteria. Of these, 103 (58.9%) were males; the mean age was 8.16 ± 3.49 years. Patient demographic characteristics are shown in Table 1. Of the 175 children with mild TBI, 15 patients (8.6%) experienced PHI (Table 2). Falling when standing/walking (*n* = 85, 48.6%) was the leading cause of injury, followed by traffic accidents (*n* = 67, 38.3%). In total, 31 patients (17.7%) had documented LOC, 97 (55.4%) had a history of vomiting, and 3 (1.7%) had a history of post-traumatic seizure. Head CT findings on admission were normal in 33 patients; they identified isolated linear skull fractures (ISFs) in 49, epidural hemorrhage (EDH) in 47, subdural hemorrhage in 11, subarachnoid hemorrhage in 16, and cerebral contusion in 19. The PHI and non-PHI group were composed of 15 and 160 patients, respectively. When the two groups were compared, univariate analysis showed that PHI was significantly correlated with the GCS score on admission, causes of head injury, vomiting, seizures, and type of TBI (all *P* < 0.05) (Table 1). timing of initial CT were 6.0 ± 7.1 and 7.4 ± 8.3 h in the PHI and non-PHI group, respectively; thus, it was shorter in the PHI group, but the difference was not significant (*P* = 0.459). We found no significant difference in the causes of injury between the PHI group and non-PHI group (*P* = 0.215), but traffic accidents were the leading cause of PHI (7 of 67, 10.4%). Moreover, neither sex nor LOC was significantly associated with PHI. Of the various potential risk factors, logistic regression identified four predictors (Table 3). Multivariate logistic regression analysis revealed that GCS scores ≥13 and EDH during the early phase of injury were independently associated with PHI (hazard ratio = 0.131, *P* = 0.018; hazard ratio = 6.612, *P* = 0.027, respectively).

**Table 1:** Clinical characteristics of the study patients.

Characteristics	PHI		*P*-value
	Yes (*n* = 15)	No (*n* = 160)	
Age (years)	9.3 (±2.4)	8.0 (±3.6)	0.198
Sex			
Female	9	63	0.102
Male	6	97	
GCS scores on admission			
15	10	146	0.010
14	1	8	
13	4	6	
Causes of injury			
Traffic accidents	7	60	0.215
Fall from standing/walking	5	80	
Fall from elevation	3	11	
Other	0	9	
Timing of initial CT (h)	6.0 ± 7.1	7.4 ± 8.3	0.459
Loss of consciousness (LOC)			
Yes	4	27	0.551
No	11	133	
Vomiting			
Yes	13	84	0.013
No	2	76	
Seizure			
Yes	2	1	0.020
No	13	159	
Type of TBI			
CT normal	1	32	0.011
ISF	1	48	
EDH	10	37	
SDH/SAH/CC	3	43	

*t-Test for age, Mann–Whitney test for GCS scores on admission, Pearson’s χ^2^ test for sex, GCS scores on admission, causes of injury, LOC, vomiting, seizure, and type of TBI were used*.

*CT, computed tomography; GCS, Glasgow Coma Scale; TBI, traumatic brain injury; ISF, isolated linear skull fractures; EDH, epidural hemorrhage; SDH, subdural hemorrhage; SAH, subarachnoid hemorrhage; CC, cerebral contusion; PHI, progressive hemorrhagic injury*.

**Table 2:** Characteristics of patients who experienced progressive hemorrhagic injury.

Age (years)	Initial GCS	Causes	Symptoms	Initial CT scan	Repeat CT scan	Treatment	Outcome
5–6	13	Traffic accident	Vomiting	EDH Rt T-P	Enlargement	NOP	Good
11–12	13	Fall	Vomiting	Bifrontal CC	Enlargement	NOP	Good
11–12	13	Traffic accident	LOC and vomiting	EDH Lt T-P	Enlargement	NOP	Good
5–6	13	Fall	Vomiting	EDH Lt T-P	Enlargement	OP	Good
11–12	14	Traffic accident	Vomiting and seizure	EDH Lt T	Enlargement	NOP	Good
7–8	15	Traffic accident	Vomiting	EDH Rt T	Enlargement	NOP	Good
9–10	15	Traffic accident	LOC and vomiting	Normal	New EDH Rt P	NOP	Good
9–10	15	Fall	LOC and vomiting	SDH Rt T	Enlargement	NOP	Good
9–10	15	Traffic accident	None	EDH Rt T	Enlargement	NOP	Good
11–12	15	Fall	Vomiting	SF Lt O	New EDH Rt O	NOP	Good
7–8	15	Traffic accident	Vomiting	EDH Rt O	Enlargement	NOP	Good
7–8	15	Fall	Vomiting	EDH Lt O	Enlargement	NOP	Good
7–8	15	Fall	LOC and vomiting	EDH Rt FT	Enlargement	NOP	Good
7–8	15	Fall	None	CC Lt P	Enlargement	NOP	Good
13–14	15	Fall	Seizure	EDH Rt F	Enlargement	OP	Good

*GCS, Glasgow Coma Scale; LOC, loss of consciousness; CT, computed tomography; EDH, epidural hemorrhage; SDH, subdural hemorrhage; CC, cerebral contusion; SF, skull fracture; Rt, right; Lt, left; F, frontal; T, temporal; P, parietal; O, occipital; OP, operation; NOP, non-operation*.

**Table 3:** Multivariate logistic regression for risk factors of progressive hemorrhagic injury.

Factors	OR	95% CI	*P*-value
GCS scores on admission			
GCS 13	0.131	0.024–0.704	0.018
GCS 14	0.098	0.004–2.403	0.155
Vomiting	0.220	0.044–1.109	0.066
Seizure	8.393	0.548–128.633	0.127
Types of TBI			
EDH	6.612	1.246–35.094	0.027
SDH	2.195	0.152–31.686	0.564
SAH	0	0.000– +∞	0.999
CC	2.955	0.348–25.130	0.321

*OR, odds ratio; CI, confidence interval; GCS, Glasgow Coma Scale; TBI, traumatic brain injury; ISF, isolated linear skull fractures; EDH, epidural hemorrhage; SDH, subdural hemorrhage; SAH, subarachnoid hemorrhage; CC, cerebral contusion*.

## Discussion

The primary concern when treating patients with TBI is the development of a clinically significant brain injury that must be rapidly identified and treated in a timely manner. As is true of all injuries, prevention is the most powerful tool when combating TBI. Once TBI occurs, however, management is centered on prevention of secondary damage to the brain (1). PHI, a secondary process, is one of the most important and devastating complications after TBI. Because of different evaluation and enrollment criteria, the reported incidences of PHI at different research centers ranged from 8 to 67% (4, 5, 12–14). In the present study, we found that PHI occurred in 15 patients (8.6%), and 2 (1.1%) underwent delayed surgical interventions. Many studies have sought to use PHI-related parameters to define threshold values for predicting PHI in patients with TBI. Several factors have been implicated as PHI determinants, including a medical history of hypertension (13), older age (14, 15), longer time from injury to the first CT scan (16), trauma severity (17), volume of the initial hematoma (17), and coagulopathy (15, 16). These studies focused principally on moderate-to-severe traumatic brain injuries in adults. However, to the best of our knowledge, few efforts have been made to predict PHI after children are admitted with mild TBI. The traumatic pathology of children is completely different from that of adults. This is because child TBI exhibits several distinctive characteristics that differ from those of adults; these differences are attributable to age-related anatomical and physiological differences, the pattern of injury based on the physical activities of children, and the difficulties associated with neurological evaluation in children. Thus, extrapolating results from adult head trauma studies to child populations may not be appropriate from the viewpoint of clinical decision-making. Therefore, the ultimate goal of our study was to assess the utility of admission data in predicting PHI in children with acute mild TBI.

We focused on possible risk factors for PHI, including causes of injury, type of TBI, and GCS scores on admission. Univariate analysis showed that causes of injury exhibited no association with PHI. However, we had only limited data on the severity of trauma, such as the heights of falls and the velocities of vehicles involved in accidents. To obtain more precise data, such factors should be considered.

Previous studies focused on patients with moderate and severe TBI (GCS < 13) and found that a lower GCS score was a risk factor for PHI, which is consistent with our study (12, 18). We found that a GCS score of 13 (hazard ratio = 0.131, *P* = 0.018) was independently associated with PHI in children with mild TBI.

As shown above, PHI was strongly associated with the type of TBI. Similar to previous studies, we found that children with mild TBI yielding normal cranial CT scans (19, 20) or exhibiting ISFs (6, 21, 22) were at very low risk for PHI and at an extremely low risk of neurosurgical intervention. In our present study, 33 patients with initial normal cranial CT scans and 49 with ISFs underwent repeat CT scans, and 1 patient in each group developed PHI. Neither required neurosurgical intervention, and neither developed sequelae. According to the literature, when initial head CT reveals no intracranial hemorrhage, the recent trend has been to monitor the child clinically. Repeat imaging is not necessary for many children, who can be safely discharged (after the emergency department evaluates the reliability of caretakers) into a safe environment with strict discharge instructions, including indications for a return to hospital.

In line with previous studies, we found that patients with EDHs (hazard ratio = 6.612, *P* = 0.027) were more prone to develop PHI than were patients with other types of lesions. Howe et al. (23) argued that repeat CT in children not exhibiting clinical deterioration should be avoided, whereas those with EDH were more likely to progress and thus required routine repeat CT, which was indicated in such cases. Kim et al. (18) found that children with EDH were at risk of PHI. A trend indicating the need for repeat CT surveillance in those with EDHs is evident due to the possibility of progression without symptoms and sudden, rapid neurological decline (23). In our present study, 15 patients experienced PHI; 10 of these patients developed acute EDH; and 2 underwent surgical treatment despite the fact that they were clinically stable. Given the high incidence of PHI caused by EDH in this population, potential predictors of a poor outcome, and early intervention may improve prognosis. EDH may represent a unique form of posttraumatic intracranial hematoma in such populations. At our institution, we schedule second routine CT scans for this subgroup of injured children.

## Conclusion

We found that a GCS score of 13 and EDH significantly increased the risk of PHI in children <15 years of age with mild TBI. These factors should be considered as indications for repeat CT examination.

## Ethics Statement

This retrospective study was approved by the Institutional Ethical Board of the Yijishan Hospital of Wannan Medical College.

## Author Contributions

Hongyi Liu and XJ designed the experiments; SC, YD and ZW performed data analysis; Hua Liu provided scientific expertise; GD wrote the manuscript. Because GD and Hua Liu contributed equally to this work, they are considered as co-first authors.

## Conflict of Interest Statement

The authors declare that the research was conducted in the absence of any commercial or financial relationships that could be construed as a potential conflict of interest.
